# Two Cases of Complete Closing up of the Glottis, One by the Growth of Two Hydatids, the Other from a Division between the Glottis and Epiglottis

**Published:** 1809-08

**Authors:** M. Delorme

**Affiliations:** Naval Surgeon


*Two Cases of complete closing up of the Glottis, one by the Growth of two Hydatids, the other from a Division between the Glottis and Epiglottis.*

Communicated to the Medical Society of Paris by M. DELORME, Naval Surgeon; and extracted from the Journal de Medicin, tom. xxxii. p. 148.

P. JOLY, captain of the galley slaves at Brest, a native of Angers, aged 44, of a bilious and dry temperament, twelve years in the service, was seized in 1802 with blenorrhagia, blenorrhagia, of which he was perfectly cured. He had the jaundice twice, from which he also recovered. In May 1807, he was seized with a low fever, followed by a tendency to ascites. A digitalis tincture was prescribed, and on the 1st of June the cure was complete. On the 13th of the same month, Joly was once more brought into the hospital, and placed under M. Delorme's care. He had been ill for nine or ten days, and exhibited the following symptoms: oppressed and sonorous respiration during sleep, which however was sound; the noise greater in the act of inspiration than in respiration; face yellow, eyes sunk; sclerotic coat slightly tinged with yellow; lips pale and thick; tongue broad, marked longitudinally from the base with yellowish streaks; skin dry and warm; pulse feeble. Although Joly experienced some obstruction in the larynx, without pain or heat, nothing was to be seen externally, nor in the lower part of the mouth. This obstruction, which slowly increased, was felt during the day, for the first time; and with the exception of constipation, the usual animal functions exhibited no derangement.

June 14th, being the eleventh day of the disease, a mineral water and a ptysan of barley and oxymel were prescribed—several vomitings of bilious matter; two stools.

15th, Sleep profound; respiration so wheezing, that it was heard at the Court Yard of the *Hospital*; deglutition difficult, particularly of liquids; infusion of borage with oxymel; electuary with ipecacuanha.

16th, M. Duret saw the patient, and having seen nothing in the pharynx, he prescribed two drachms of acetate of ammonia in a pint of ptysan.

17th, The same noise in breathing; sleep sound; prescription the same—difficulty of swallowing; a powerful blister applied to the nape of the neck.

18th, Scarification of the eschar; nothing new; the noise in breathing is less when the patient is awake.

19th, One grain of extract of opium in two pills, to excite perspiration.

20th, Noise in breathing less; a bad appetite; nothing particular until the 10th of July, and during this interval there were three or four days without any noise in breathing; but this symptom returned on the above day.

July 23d, The patient complained of nothing but an obstruction in the larynx.

24th, Being the fortieth day of the disease, Joly was found dead in his bed.

During the last stage of the disease, the pulse was almost always feeble, but regular; the patient retained his senses to the last, and his appetite was good until the 7th of July. No obstruction was experienced except in swallowing of liquids, which constantly produced violent coughing.

### Dissection of the Body.

Face and lips violet coloured, frothy mucus in the mouth; nothing particular was observed in the abdomen, with the exception of nineteen lumbrical living worms in the jejunum and illium; the lungs were only a little flabby, and of their natural colour; a veinous turgescence of the right cavities of the heart; the left were almost empty; the brain perfectly sound, as well as its membranes.

Incisions from the lips to the condyles, which were sawed through, facilitated the inspection of the lower part of the mouth, in which nothing particular was observed. Incisions were afterwards made as far as the spine, into all the soft parts situated before and behind, above the sternum, without in the least changing the connection between the tracheal artery and the œsophagus; and every thing was detached from the jaw including the tongue. By this means we saw, behind and under the epiglottis, near its junction with the posterior part of the larynx, two semi-transparent vesicles, oval both behind and before, as large as a small nut, fixed against each other, and occupying (the right one in particular) a part of the ventricles, from which they proceeded towards the epiglottic ligaments; the side not attached was turgid, both before and behind, and they entirely closed up the glottis. One of the assistants inadvertently placed his finger upon one of the vesicles and burst it, when a clear thready matter issued similar to albumen; the division of the posterior part of the œsophagus, admitted of our discovering the whole extent of the disease.

The Council of the Board for superintending the Health of the French Navy, ordered a drawing to be made of this dissection. M. Duret thought that if a similar case again occurred, great advantages might be expected from the section of the crico-thyröid membrane, cutting a little into the thyröid cartilage. In fact, he performed this operation, and the separation of the edges of the section exhibited the two vesicles, which might have been easily removed from the living subject, if their existence had been suspected. In the above case, the cure would have been the the more certain, as the whole tracheal artery was very sound.

The hydatid preserved was shining, smooth, semi-transparent, and more consistent than the other; it adhered to the adjoining parts by the half of its external circumference; in front it was detached, and merely touched the other at its internal edge. It contained a limpid fluid, in which a whitish flake floated, similar to what is found in the white of a new laid egg, but much smaller; it occupied the anterior part of the vesicle, and exhibited a brown point in its centre, as large as the point of a small pin; the animal organization of which could not be ascertained. The liquor was tasteless and insipid; it gave a turbid appearance to the acetous acid, and to alcohol, but there were no flakes; the whitish substance was only a little hardened; when held in a spoon over the flame of a candle, the liquor remained fluid in the centre, but was coagulated at the sides.

The above account is interesting in a double point of view; both from the accurate manner in which it is drawn up, and from the difficulty of discovering the cause of a similar affection, should it again occur. Unique of its kind, it deserves to be recorded, in order to enlarge the mass of insulated facts, which may one day be referred to their proper causes. On opening the body of a patient like M. Joly, it is not sufficient to ascertain that by such and such methods a hydatid may be extirpated, but the real object is, to ascertain its actual existence, and the precise spot which it occupies.

M. Delorme judiciously observes, that there was in this case no pathognomical sign, and that all the symptoms might have been referred to any other affection of the same part, the croup for example, if the wheezing respiration only was regarded. As to the patient in question, it ought to be remarked, that no convulsive cough made its appearance, and that there was no threatened suffocation, nor any of those strong symptoms which indicate croup beyond the possibility of mistaking it. If the opening of the body shewed what might have been successfully practised, it also accounts for the inadequacy of the remedies employed, and there is nothing wonderful in the patient's sudden death. The veinous turgidity of the right cavities of the heart, accounts for the size of the lips, the tongue, &c. There ought also to have been a similar swelling of the veins of the head, and a collection of serous matter under the cranium; at least, this circumstance is

 probable probable, although it is positively asserted, that these parts presented nothing particular.

The sudden death of M. Joly brings to my recollection, a case, which I think ought to be generally known. In May, 1795, two chimney-sweeps, about 13 or 14 years of age, were on their way to the country. One of them had eleven louis d'ors in his pocket, and he fell asleep on the road. His companion robbed him after cutting his throat. The unfortunate victim when brought to the Hotel-Dieu, at Paris, had a transverse wound between the os hyoides, and the thyroid cartilage; every thing shewed that there was a communication from the external to the internal part of the pharynx. Under the care of M. Pelletan, the cicatrix was soon almost entirely healed up. One day, in the interval between the dressings of the wound, the patient was suddenly ordered to attend the judge, to give evidence against the perpetrator of the crime. He had scarcely descended the staircase, when he fell down dead.

On opening the body, we found the left side of the epiglottis detached from the glottis, and from the root of the tongue; it had fallen across the glottis, so as suddenly to suspend respiration. Who could have foreseen such an accident?

